# Detection of distant metastases in patients with oesophageal or gastric cardia cancer: a diagnostic decision analysis

**DOI:** 10.1038/sj.bjc.6603960

**Published:** 2007-09-11

**Authors:** E P M van Vliet, E W Steyerberg, M J C Eijkemans, E J Kuipers, P D Siersema

**Affiliations:** 1 Department of Gastroenterology and Hepatology, Erasmus MC – University Medical Center Rotterdam, Rotterdam, The Netherlands; 2 Department of Public Health, Erasmus MC – University Medical Center Rotterdam, Rotterdam, The Netherlands

**Keywords:** staging, oesophageal cancer, metastases

## Abstract

Computed tomography (CT) is presently a standard procedure for the detection of distant metastases in patients with oesophageal or gastric cardia cancer. We aimed to determine the additional diagnostic value of alternative staging investigations. We included 569 oesophageal or gastric cardia cancer patients who had undergone CT neck/thorax/abdomen, ultrasound (US) abdomen, US neck, endoscopic ultrasonography (EUS), and/or chest X-ray for staging. Sensitivity and specificity were first determined at an organ level (results of investigations, i.e., CT, US abdomen, US neck, EUS, and chest X-ray, per organ), and then at a patient level (results for combinations of investigations), considering that the detection of distant metastases is a contraindication to surgery. For this, we compared three strategies for each organ: CT alone, CT plus another investigation if CT was negative for metastases (one-positive scenario), and CT plus another investigation if CT was positive, but requiring that both were positive for a final positive result (two-positive scenario). In addition, costs, life expectancy and quality adjusted life years (QALYs) were compared between different diagnostic strategies. CT showed sensitivities for detecting metastases in celiac lymph nodes, liver and lung of 69, 73, and 90%, respectively, which was higher than the sensitivities of US abdomen (44% for celiac lymph nodes and 65% for liver metastases), EUS (38% for celiac lymph nodes), and chest X-ray (68% for lung metastases). In contrast, US neck showed a higher sensitivity for the detection of malignant supraclavicular lymph nodes than CT (85 *vs* 28%). At a patient level, sensitivity for detecting distant metastases was 66% and specificity was 95% if only CT was performed. A higher sensitivity (86%) was achieved when US neck was added to CT (one-positive scenario), at the same specificity (95%). This strategy resulted in lower costs compared to CT only, at an almost similar (quality adjusted) life expectancy. Slightly higher specificities (97–99%) were achieved if liver and/or lung metastases found on CT, were confirmed by US abdomen or chest X-ray, respectively (two-positive scenario). These strategies had only slightly higher QALYs, but substantially higher costs. The combination of CT neck/thorax/abdomen and US neck was most cost-effective for the detection of metastases in patients with oesophageal or gastric cardia cancer, whereas the performance of CT only had a lower sensitivity for metastases detection and higher costs. The role of EUS seems limited, which may be due to the low number of M1b celiac lymph nodes detected in this series. It remains to be determined whether the application of positron emission tomography will further increase sensitivities and specificities of metastases detection without jeopardising costs and QALYs.

Patients with oesophageal or gastric cardia cancer have a dismal prognosis, due to the presence of locally advanced cancer, lymph node metastases or distant metastases at the time of presentation in more than 50% of patients (Lightdale, 1999). Investigations that can be used for staging oesophageal or gastric cardia cancer include endoscopic ultrasonography (EUS) (Vickers and Alderson, 1998), computed tomography (CT) of neck, thorax, and abdomen (Maerz *et al*, 1993), ultrasound (US) of the neck (Griffith *et al*, 2000) and abdomen (van Overhagen *et al*, 1992), chest X-ray (Stein *et al*, 2001), bronchoscopy (Riedel *et al*, 1998), and ^18^F-fluoro-2-deoxy-D-glucose positron emission tomography (FDG-PET) (Leccisotti, 2006). The TNM stage of patients with oesophageal or gastric cardia cancer is usually established by a combination of these investigations. The TNM system is subdivided into the T stage describing the extent of local invasion of the tumour through the oesophageal wall, the N stage indicating whether metastases are present in regional lymph nodes, and the M stage describing whether distant metastases are present (Fleming *et al*. 1997).

The presence of distant metastases from oesophageal or gastric cardia cancer is usually investigated by more than one modality. In almost all patients, CT neck/thorax/abdomen is a standard investigation. It is however not clear whether EUS, US neck and/or abdomen, and chest X-ray are also necessary for assessing the presence of distant metastases in these patients. In this study, we aimed to determine the diagnostic value of EUS, US abdomen, US neck, and chest X-ray in addition to CT in patients with oesophageal or gastric cardia cancer. We evaluated these diagnostic procedures both at an organ level and at a patient level for the detection of metastases. The assumption was that the finding of distant metastases in patients with oesophageal or gastric cardia cancer would eliminate the option of a curative surgical treatment.

## PATIENTS AND METHODS

### Patients

We used a prospectively collected database with information on 1088 patients with oesophageal or gastric cardia cancer who were diagnosed and treated between January 1994 and October 2003 at the Erasmus MC – University Medical Center Rotterdam, The Netherlands. Data that were collected included general patient characteristics, results of staging investigations, treatment modalities, and postoperative TNM stage. Additional information, which was not present in the database but necessary for this study, was obtained from the electronic hospital information system. We assessed which preoperative investigations had been performed in these 1088 patients.

**Figure 1:**
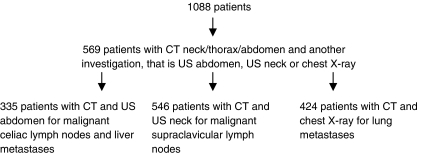
Flow diagram of inclusion of patients.

In 906/1088 (83%) patients, oesophageal or gastric cardia cancer was first diagnosed in a regional centre and, subsequently, these patients were referred to our referral centre. Patients often underwent preoperative staging investigations in these regional centres; however, the results of these investigations were not included in our analyses. In contrast, we identified 569 oesophageal or gastric cardia cancer patients who had undergone CT neck/thorax/abdomen and at least one other investigation, that is, US abdomen, US neck and/or chest X-ray, in our centre (Figure 1). Some of these patients had also undergone EUS (see below). The reasons for performing these additional staging investigations were in most cases a CT that was negative for the presence of metastases or the suspicion of metastases on CT for which additional evidence was required. FDG-PET was not performed in our centre during the study period (1994–2003), and therefore, the additional value of this modality could not be determined in this study.

### Staging investigations

The organs to which oesophageal or gastric cardia cancers most frequently metastasise, that is, liver, celiac lymph nodes, supraclavicular lymph nodes, and lung, were first evaluated separately (‘organ level’). For this, we assessed whether both CT and US abdomen, if indicated with fine-needle aspiration (FNA), should be performed for the detection of liver metastases using the results of 335 patients who had undergone both investigations. In addition, for the detection of malignant celiac lymph nodes, we analysed 143 patients who had undergone CT, US abdomen, and EUS, for malignant supraclavicular lymph nodes, 546 patients who had undergone CT and US neck, if indicated with FNA and for lung metastases, 424 patients who had undergone CT and chest X-ray (Figure 1). In case of a suspicious lesion, FNA was performed if the result could change the treatment decision. If multiple suspicious lesions were present, FNA of the most suspicious lesion was performed. The results of the investigations were compared with the gold standard, which was postoperative pathological TNM stage, result of FNA, or a radiological finding in the relevant organ with ⩾6 months of follow-up. In patients in whom CT was positive, however, US neck or abdomen negative, the latter was repeated to determine whether the lesion could be found using the CT information and to evaluate whether FNA could be performed. In the current study, we did not use the results of this repeated investigation, but used the result of the initial US neck or abdomen. Nevertheless, if FNA could be performed, the FNA result was used as gold standard.

For the interpretation of the results on a patient level, we considered celiac lymph node metastases as regional (N1) if the primary tumour was located in the gastric cardia, as stage M1a if the tumour was located in the distal part of the oesophagus and as stage M1b if the tumour was located in the mid or proximal part of the oesophagus (Thompson, 1997). As oesophageal cancers with M1a celiac lymph node metastases in many centres are considered to be resectable (Hagen *et al*, 2001), only M1b celiac lymph nodes were considered to be distant metastases in the part of the study that was related to the interpretation on patient level. In our data, only three patients had M1b celiac lymph nodes. In addition, malignant supraclavicular lymph nodes were considered as N1 if the tumour was located in the proximal part of the oesophagus and as M1b if the tumour was located in the mid or distal part of the oesophagus or in the gastric cardia (Thompson, 1997).

### Statistical analyses

Sensitivities, specificities, false-positive and false-negative results of CT, US abdomen, EUS, US neck, and chest X-ray, alone or in combination, for the detection of metastases in the various organs were calculated. The combined results were calculated twice. First, the result was considered positive for metastases if at least one of two investigations that were performed for a particular organ was positive, and negative if both investigations were negative (one-positive scenario). This is a strategy that uses the possible additional diagnostic information of the second investigation in case of a negative CT. If the CT is positive, the result of another investigation is irrelevant in this strategy, because the final result will remain positive irrespective of the result of the other investigation. Second, the result was considered positive if both CT and another investigation were positive and negative if at least one of the investigations was negative (two-positive scenario). This is a strategy that uses additional diagnostic investigations to confirm a positive CT finding. If the CT is negative, the performance of another investigation is unnecessary using this strategy, because the final result will remain negative irrespective of the result of the other investigation. For celiac lymph nodes, the number of false-positive and false-negative results was also calculated for the combination of CT, US abdomen, and EUS.

In addition to analyses at the organ level, we considered analyses at the patient level. Here, we assessed whether distant metastases (M1b) were present in liver, lung, celiac lymph nodes, and supraclavicular lymph nodes and, consequently, whether a curative oesophageal resection should have been performed or not on the basis of combinations of staging investigations using the data of 264 patients who had undergone all investigations. The assumption was that an oesophageal resection should only be performed if no distant metastases are detected. Similarly to the analyses at the organ level, the strategies included CT, and the one-positive and two-positive scenarios for the detection of metastases in liver, celiac lymph nodes, supraclavicular lymph nodes, and lung. In total, 81 different combinations of investigations were possible (3 strategies for 4 organs). Sensitivities and specificities for the detection of distant metastases at the patient level were calculated for each combination.

Of the 569 patients, 305 patients had one or more missing values, that is, these patients had not undergone all staging investigations. An exploratory analysis was performed in which missing values were imputed for these 305 patients by the expectation maximisation (EM) method as implemented in SPSS software (version 12, SPSS Inc., Chicago, IL, USA). This was repeated five times to incorporate uncertainties in the imputation process. Sensitivities and specificities for the detection of distant metastases were calculated for each combination of investigations using the five completed data sets (Rubin and Little, 2002; Schafer and Graham, 2002).

We plotted sensitivity against one-specificity in a receiver operating characteristic (ROC) curve for a visual comparison of the accuracy of combinations of staging investigations using the data of 264 patients who had undergone all investigations. Sensitivity is the proportion of patients who are correctly identified as having distant metastases (true positive results), and one-specificity is the proportion of patients in whom the gold standard is negative for distant metastases, and who are incorrectly identified as positive by the staging investigation (false-positive results). ROC curves were made for the detection of distant metastases (M1b) with CT and the combination of CT and another investigation (both the two-positive and one-positive scenarios) in an organ, whereas in the other organs we only included the CT result. For example, to assess whether both CT and US abdomen should be performed to determine whether liver metastases were present, we compared three different strategies: (1) combination of CT and US abdomen in the two-positive scenario for the liver and CT for the other organs; (2) combination of CT and US abdomen in the one-positive scenario for the liver and CT for the other organs; (3) CT for all organs.

The McNemar test was performed to determine whether the differences between sensitivities of pairs of tests and specificities of pairs of tests were statistically significant. We calculated accuracy rates and 95% confidence intervals using exact methods (Knottnerus, 2001). All *P*-values were based on two-sided tests of significance. A *P*-value<0.05 was considered as statistically significant.

### Cost-effectiveness analysis

Costs, life expectancies and quality-adjusted life years (QALYs) were compared between the different combinations of investigations. As an extreme policy, we considered that all patients could undergo surgery. Costs were estimated from data of the Erasmus MC – University Medical Center Rotterdam, The Netherlands. The extra costs of a resection over palliative treatment were estimated to be approximately $50 000, and the costs for the performance of diagnostic investigations were for US: $100; for chest X-ray: $60; and for CT: $750. These diagnostic work-up costs were negligible compared to the costs of resection, and were therefore not taken into account. Life expectancy and QALYs were taken from a previous study (Wallace *et al*, 2002). Life expectancy was assumed to be 2.41 and 1.00 year for local/regional disease with and without resection, respectively, and 0.42 and 0.37 year for distant disease with and without resection, respectively. QALYs were estimated to be 1.45 and 0.70 for local/regional disease, and 0.17 and 0.19 for distant disease, with and without resection, respectively. A cost-effectiveness plane was constructed in which the differences in costs between strategies (Δ costs) were plotted against the differences in QALY (Δ QALY). Costs were expressed per $1000 (k$) for easier interpretation.

## RESULTS

In Table 1, patient and tumour characteristics are shown for all 569 patients who had undergone both CT neck/thorax/abdomen and at least one other investigation, that is, US abdomen, US neck and/or chest X-ray, for the 264 patients who had undergone all investigations and for the 305 patients who had undergone some diagnostic investigations. χ^2^ testing revealed that the differences between the patients with all (*n*=264) or some (*n*=305) diagnostic investigations were statistically not significant.

### Organ level

In Table 2, the gold standard diagnoses are shown per organ. Positive gold standard diagnoses were confirmed by FNA or resection in the majority of cases (92/135, 68%), whereas such confirmation could not be used in the remaining cases. A reason for this was that several patients had two or more suspicious lesions and FNA had already been performed for one of these lesions, which confirmed the presence of a distant metastasis. FNA of the other suspicious lesions was therefore not indicated in these patients.

Sensitivity for the detection of liver metastases was higher for CT than for US abdomen, but this was statistically not significant (73 *vs* 65%, *P*=0.63; Table 3). Sensitivity for celiac lymph node metastases was higher for CT than for US abdomen (69 *vs* 44%, *P*=0.08) and for EUS (38%, *P*=0.03). Sensitivity for supraclavicular lymph node metastases was higher for US neck than for CT (85 *vs* 28%, *P*<0.001). Sensitivity for lung metastases was slightly higher for CT than for chest X-ray, but this was statistically not significant (90 *vs* 68%, *P*=0.29).

Accuracies for combinations of staging investigations all exceeded 80% (Table 3). If only CT was performed for liver metastases, the number of false-positive results was 10 and the number of false-negative results was 7. The addition of US abdomen (one-positive scenario) resulted in a decline in the number of false-negative results to 6, with also 10 false-positive results. For celiac lymph nodes, the combination of CT *plus* US abdomen (one-positive scenario) resulted in fewer false-negative results in comparison with the performance of CT alone (6 *vs* 10). If only CT was performed for supraclavicular lymph nodes, the number of false-negative results was 42. With US neck or the combination of CT and US neck (one-positive scenario) fewer false-negative results were obtained (9 and 8, respectively). Overall, the numbers of false-positive results were higher than the number of false-negative results for combinations of CT with another investigation in the one-positive scenarios. In contrast, the number of false-negative results was higher in the two-positive scenarios (Table 3).

### Patient level

On the organ level, the results of EUS for the detection of malignant celiac lymph nodes were inferior than for CT and US abdomen. For that reason, EUS was considered to be less relevant for the detection of distant metastases, and was not included in the part of the analyses concerning patient level.

**Figure 2:**
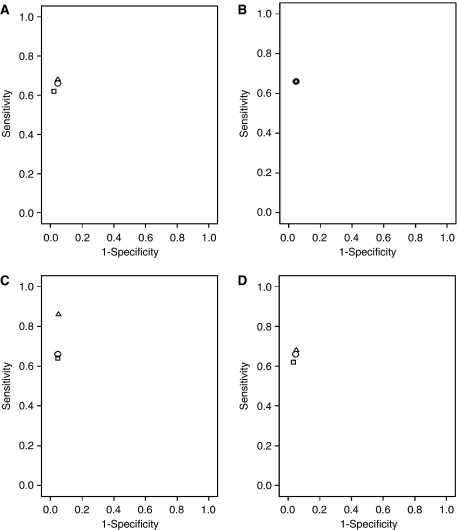
ROC curves for the detection of metastases with CT and the combination of CT and another investigation (one-positive and two-positive scenario) in an organ, whereas for the other organs only the result of CT was included *vs* the gold standard, with (**A**) liver, (**B**) celiac lymph nodes, (**C**) supraclavicular lymph nodes, and (**D**) lung. ○, CT for all regions; △, combination of CT and another investigation for the investigated region, with a positive result if at least one investigation is positive (one-positive), and CT for the other regions; □, combination of CT and another investigation for the investigated region, with a positive result if both investigations are positive (two-positive), and CT for the other regions.

In the ROC curve, sensitivity and specificity of CT and the combinations of CT and US abdomen (two-positive and one-positive scenario) were more or less equal for liver metastases (Figure 2A), which was in line with the results at the organ level. Adding US abdomen (two-positive and one-positive scenario) to CT did not result in a difference in sensitivity and specificity for malignant celiac lymph nodes (Figure 2B). For malignant supraclavicular lymph nodes, the combination of CT and US neck (one-positive scenario) resulted in a better overall sensitivity compared to CT alone and the combination of CT and US neck (two-positive scenario), whereas specificities were comparable (Figure 2C). For lung metastases, sensitivities and specificities were roughly equal across the strategies (Figure 2D).

The sensitivity for detecting distant metastases was 66% and specificity was 95% if only CT was performed for all organs (Table 4). Higher sensitivities and specificities could be obtained by the addition of one or more other staging investigations. The highest sensitivity, which could be obtained with 12 of the 81 different combinations of staging investigations, was 86%. For 6 of these 12 combinations, the specificity was 94.4%, whereas for 6 other combinations the specificity was only slightly higher (94.9%). The lowest number of investigations for a sensitivity of 86% and a specificity of 94.9% was the combination of CT *plus* US neck for the detection of supraclavicular lymph node metastases (one-positive scenario), and CT only for the detection of metastases in celiac lymph nodes, liver, and lung. A slightly higher specificity of 97% was achieved by the addition of US abdomen for liver metastases, but only in the two-positive scenario. When chest X-ray (two-positive scenario) for the detection of lung metastases was added, the specificity further increased to 99%. Sensitivity declined with increasing specificity, meaning that more patients would have undergone a curative treatment option in the presence of distant metastases (more false-negative results). The addition of US abdomen for the detection of malignant celiac lymph nodes did not result in better results; however, only 3/264 patients had M1b celiac lymph nodes, whereas 49 other patients had M1a celiac lymph nodes that did not preclude a resection.

The average results obtained from the data with imputation of missing values (*n*=569) were roughly equal compared to the results obtained from the complete data of patients who had undergone all staging investigations (*n*=264 patients; Table 4).

**Figure 3:**
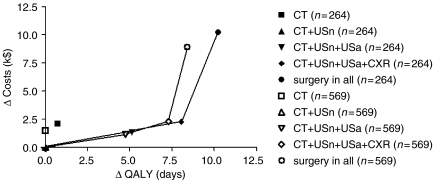
Marginal cost-effectiveness plane calculated in patients with oesophageal or gastric cardia cancer who had undergone all staging investigations (*n*=264) and using the five completed data sets (*n*=569). The combination of CT and US neck for the detection of supraclavicular lymph node metastases (one-positive scenario), and CT only for the detection of metastases in celiac lymph nodes, liver and lung was considered as reference strategy. CT=computed tomography; CXR=chest X-ray; QALY, quality adjusted life year; USa=ultrasound abdomen; USn=ultrasound neck.

If only CT would have been performed, costs were high and QALYs were low compared to other combinations of investigations. Therefore, the performance of CT only was dominated by other combinations of investigations. Costs were lowest for the combination of CT and US neck for supraclavicular lymph node metastases (one-positive scenario) and CT only for the other organs (average costs per patient: 39.8k$; Table 5; Figure 3).

## DISCUSSION

Surgery is presently the only established curative treatment option for patients with oesophageal or gastric cardia cancer. However, surgery is invasive, with a substantial risk of morbidity and mortality. Therefore, adequate staging is of outmost importance to select patients without distant metastases for undergoing surgery. In this study, we assessed which traditional staging investigations should be performed in patients with oesophageal or gastric cardia cancer to determine whether distant metastases were present and, consequently, whether a curative treatment, that is, an oesophageal resection, could be performed. Our findings demonstrated that the performance of CT only was not sensitive enough for the detection of distant metastases. The addition of US neck to CT for the detection of supraclavicular lymph node metastases resulted in the highest sensitivity. For a slightly higher specificity (less false positives), US abdomen and chest X-ray could be added, but this required that both CT and these investigations were positive for metastases to define the result as positive (two-positive scenario). A higher specificity would however result in a decline in sensitivity and consequently in more resections in patients with distant metastases. We recognise that the requirement of two staging procedures being positive is not a common clinical strategy. Nonetheless, another investigation, in addition to CT, is sometimes already used to confirm the suspicion of metastases on CT.

The choice for the optimal combination of investigations usually depends on the relative weight one is willing to accept for the number of patients with a false-positive (no curative treatment option in the absence of distant metastases) *vs* those with a false-negative staging result (a curative treatment option in the presence of distant metastases). We formally assessed this balance of false-positive *vs* false-negative staging results in a cost-effectiveness analysis. A combination of investigations with a high sensitivity for detecting distant metastases, but a lower specificity, would result in relatively low costs, but the average life expectancy and average QALYs would also be relatively low (Table 5). This is due to the substantially lower QALYs for patients with local/regional disease who would not undergo a resection (false-positive staging result) compared with patients with local/regional disease undergoing a resection. A higher specificity was only achievable with a lower sensitivity, resulting in more patients undergoing a resection in the presence of distant metastases. This resulted in higher QALYs, but also in substantially higher costs. In cost-effectiveness analyses, a ratio of approximately 50k$ per QALY is generally considered to be acceptable for a clinical strategy compared to a reference strategy (Gold *et al*, 1996). The ratios of the alternatives were all far above this threshold in the present study (Table 5) and, therefore, no single combination of investigations was more cost-effective than the combination CT and US neck.

On the basis of the results on the organ level, we concluded that the performance of US abdomen, US neck, and chest X-ray, respectively, in combination with CT resulted in a higher accuracy compared to the performance of CT only. The addition of EUS had no additional value over the performance of CT plus US abdomen for the detection of malignant celiac lymph nodes, and for that reason, EUS was not included in the part of the analysis concerning patient level. We recognise that the sensitivity of EUS for the detection of celiac lymph nodes was lower in our study compared to the literature (38% *vs* 75–100%, respectively), whereas specificity was comparable (94% *vs* 50–100%, respectively) (Catalano *et al*, 1999; Eloubeidi *et al*, 2001; Vazquez-Sequeiros *et al*, 2001; Parmar *et al*, 2002). An explanation for this is probably that in patients who were diagnosed and staged in the early years of this study, FNA was not performed during EUS. In addition, dilation was often not performed in patients with a stenotic tumour. The few studies that have reported on sensitivities and specificities of EUS for the detection of celiac lymph node metastases have mainly been performed in centres with a high volume of EUS procedures and a higher level of expertise. Recently, we demonstrated that results of EUS performed in a centre where <50 EUS procedures per endoscopist per year are performed compare unfavourably with those reported from high-volume EUS centres (van Vliet *et al*, 2006b). Until 2003, endoscopists in our centre performed less than 50 EUS procedures per person per year. Since 2003, we changed this policy and presently only two dedicated EUS endoscopists with considerable annual experience (>50 EUS procedures per year) perform these procedures. The results for the detection of malignant celiac lymph nodes obtained by these endoscopists in the period between November 2003 and May 2006 were higher compared to the results reported in the previous period, with a sensitivity of 62% and a specificity of 92% (unpublished results). As the data used in the present study were obtained in patients diagnosed before November 2003, the additional value of EUS for the detection of malignant celiac lymph nodes is likely to have been underestimated in the present study. We assessed whether better EUS results would have changed the results of our study. Here, we used the median sensitivity and specificity of EUS from the literature (80 and 92%, respectively). In the data set of 264 patients who had undergone CT neck/thorax/abdomen, US abdomen, US neck, and chest X-ray, we included these reported results of EUS. This showed, however, that EUS had only limited additional value for the detection of distant metastases at the patient level. An explanation for this could, however, be that only 3/264 (1%) patients had M1b celiac lymph nodes according to the gold standard, whereas 49 other patients had M1a celiac lymph nodes that did not preclude resection. Two of the three patients with M1b celiac lymph nodes had also supraclavicular lymph node metastases that were detected by both CT and US neck, and these patients would not have undergone a resection anyhow, irrespective of the finding of M1b celiac lymph nodes by EUS or another investigation. In the present study, the role of EUS seems to be limited for the detection of distant metastases, which may be particularly due to the low number of M1b celiac lymph nodes. Nevertheless, EUS is still a useful method to determine the extent of tumour invasion through the oesophageal wall (T stage) and to investigate whether regional lymph node metastases (N stage) are present (Lightdale and Kulkarni, 2005).

There are some other limitations to our study. Patients included in this study were a selection of patients diagnosed with oesophageal or gastric cardia cancer. This study was performed in a referral centre and not all patients in whom distant metastases were detected in regional centres were referred to our centre. In addition, only preoperative staging investigations that were performed in our centre were included in this retrospective study, as it is known that the diagnostic sensitivity for metastases detection is higher for investigations made and evaluated in a high-volume referral centre compared to low-volume regional centres (van Vliet *et al*, 2006a). Furthermore, only patients who had undergone CT neck/thorax/abdomen and one or more other investigations, that is, US abdomen, US neck and/or chest X-ray, in our centre were included. However, no statistically significant differences were found within the whole group of patients (*n*=569), according to whether all or some investigations had been performed.

Second, sensitivities and specificities of CT, US abdomen, US neck, and chest X-ray (Table 3) were largely in line with the literature (Thompson *et al*, 1983; Quint *et al*, 1985; Yoshinaka *et al*, 1985; Lehr *et al*, 1988; Watt *et al*, 1989; Van Overhagen *et al*, 1993; Tachimori *et al*, 1994; Bonvalot *et al*, 1996; Chandawarkar *et al*, 1996; Catalano *et al*, 1999; Natsugoe *et al*, 1999; Reed *et al*, 1999; Eloubeidi *et al*, 2001; Vazquez-Sequeiros *et al*, 2001; Parmar *et al*, 2002; Kneist *et al*, 2003). Nevertheless, in other centres, the optimal strategy to stage patients with oesophageal or gastric cardia cancer is not automatically the combination of CT and US neck, as sensitivities and specificities of combinations of investigations largely depend on the quality of the staging investigations in a centre. This quality is determined by both experience of the investigator and quality of the equipment.

Third, positron emission tomography (PET) scanning was not used in the patients who were included in this retrospective study. PET has been suggested to be potentially valuable for the detection of distant metastases, especially modern PET-CT scans (Rosenbaum *et al*, 2006). Therefore, further studies need to determine the exact role of PET in the staging of oesophageal or gastric cardia cancer.

Finally, the relatively low numbers of patients with metastases may limit the interpretation of comparisons of sensitivities and specificities. However, the conclusion on the optimal staging strategy is quite robust, because detectable metastases in some organs are relatively rare. Extra diagnostic evaluations can hence not be very cost-effective, although no formal analysis of uncertainty was performed (bootstrapping, or construction of acceptability curves) (Willan and Briggs, 2006).

In conclusion, the combination of CT neck and US neck for the detection of supraclavicular lymph node metastases and CT thorax/abdomen for the detection of metastases in celiac lymph nodes, liver, and lung is a cost-effective strategy for the detection of distant metastases in patients with oesophageal or gastric cardia cancer. US abdomen and chest X-ray have only limited additional value in the detection of distant metastases in these patients. These staging investigations should only be performed for specific indications in patients with oesophageal or gastric cardia cancer, as the treatment decision is not improved in most of the patients if these investigations are added to the diagnostic work-up. The role of EUS for the detection of distant metastases seems also be limited, which may be particularly due to the low number of M1b celiac lymph nodes in the present study.

## Figures and Tables

**Table 1:** Patient and tumour characteristics of 569 patients who had undergone CT neck/thorax/abdomen and at least one other investigation, that is, US abdomen, US neck, and/or chest X-ray, the subgroup of 264 patients who had undergone all these investigations and the subgroup of 305 patients who had undergone CT neck/thorax/abdomen plus at least one other diagnostic investigation for oesophageal or gastric cardia cancer staging

**Variable**	***n*=569 (all patients)**	***n*=264 (CT+US neck+US abdomen+chest X-ray)**	***n*=305 (CT+⩾1 other investigation**
Mean age±standard deviation (years)	61.9±10.2	61.2±10.4	62.5±10.0
*Sex* (%)			
Male	436 (77)	207 (78)	229 (75)
Female	133 (23)	57 (22)	76 (25)
			
*Histology of tumour at biopsy* (%)			
Squamous cell carcinoma	227 (40)	116 (44)	111 (37)
Adenocarcinoma	304 (53)	132 (50)	172 (56)
Other	38 (7)	16 (6)	22 (7)
			
*Location of tumour* (%)			
Cervical	5 (1)	4 (2)	1 (1)
Upper 1/3 thoracic	30 (5)	11 (4)	19 (6)
Central 1/3 thoracic	101 (18)	53 (20)	48 (16)
Lower 1/3 thoracic	219 (38)	96 (36)	123 (40)
Gastroesophageal junction	214 (38)	100 (38)	114 (37)
			
*Distant metastases according to gold standard* (%)			
M0	473 (83)	214 (81)	259 (85)
M1	96 (17)	50 (19)	46 (15)

**Table 2:** Gold standards in 569 patients with oesophageal or gastric cardia cancer undergoing preoperative investigations for the detection of metastases

	**Gold standard**		
**Organ**	**FNA**	**Postoperative stage**	**Radiological finding with ⩾6 months of follow-up**
*Liver* (*n*=*335*)			
Positive (*n*=26)	15	0	11
Negative (*n*=309)	22	8	279
			
*Celiac lymph nodes* (*n*=*143*)			
Positive (*n*=32)	6	15	11
Negative (*n*=111)	3	74	34
			
*Supraclavicular lymph nodes* (*n*=*546*)			
Positive (*n*=58)	44	6	8
Negative (*n*=488)	68	35	385
			
*Lung* (*n*=*424*)			
Positive (*n*=19)	5	1	13
Negative (*n*=405)	7	0	398

FNA, fine-needle aspiration.

**Table 3:** Sensitivities and specificities of CT, US abdomen, EUS, US neck, and chest X-ray only and the number of false-positive and false-negative results and the accuracy rates plus 95% confidence intervals for CT, US abdomen, EUS, US neck, and chest X-ray only and the combinations of CT and the other investigations in patients with oesophageal or gastric cardia cancer

	**Sensitivity**	**Specificity**	**False-positive: false-negative**	**Accuracy rate**	**95% confidence interval**
*Liver* (*n*=*335, 26 metastases)*					
CT	19/26 (73%)	299/309 (97%)	10 : 7	0.95	0.92–0.97
USa	17/26 (65%)	308/309 (99%)	1 : 9	0.97	0.95–0.99
CT+USa: 1 positive			10 : 6	0.95	0.92–0.97
CT+USa: 2 positive			1 : 10	0.97	0.94–0.98
					
*Celiac lymph nodes* (n=*143, 32 metastases)*					
CT	22/32 (69%)	102/111 (92%)	9 : 10	0.87	0.80–0.92
USa	14/32 (44%)	111/111 (100%)	0 : 18	0.87	0.81–0.92
EUS	12/32 (38%)	104/111 (94%)	7 : 20	0.81	0.74–0.87
CT+EUS: 1 positive			11 : 6	0.88	0.82–0.93
CT+EUS: 2 positive			5 : 24	0.80	0.72–0.86
CT+USa: 1 positive			9 : 6	0.90	0.83–0.94
CT+USa: 2 positive			0 : 22	0.85	0.78–0.90
CT+USa+EUS: ⩾1 positive			11 : 5	0.89	0.82–0.93
CT+USa+EUS: ⩾2 positive			5 : 13	0.87	0.81–0.92
CT+USa+EUS: 3 positive			0 : 30	0.79	0.71–0.85
					
*Supraclavicular lymph nodes* (n=*546, 58 metastases)*					
CT	16/58 (28%)	484/488 (99%)	4 : 42	0.92	0.89–0.94
USn	49/58 (85%)	484/488 (99%)	4 : 9	0.98	0.96–0.99
CT+USn: 1 positive			7 : 8	0.97	0.96–0.98
CT+USn: 2 positive			1 : 43	0.92	0.89–0.94
					
*Lung* (n=*424, 19 metastases)*					
CT	17/19 (90%)	399/405 (99%)	6 : 2	0.98	0.96–0.99
CXR	13/19 (68%)	404/405 (99%)	1 : 6	0.98	0.97–0.99
CT+ CXR: 1 positive			7 : 0	0.98	0.97–0.99
CT+ CXR: 2 positive			0 : 8	0.98	0.96–0.99

CT=computed tomography; CXR=chest X-ray; EUS=endoscopic ultrasonography; USa=ultrasound abdomen; USn=ultrasound neck.

**Table 4:** Sensitivities and specificities for the detection of distant metastases with combinations of staging investigations in patients with oesophageal or gastric cardia cancer who had undergone all investigations (*n*=264) and the average sensitivity and specificity of the 5 completed data sets (*n*=569)

				***n*=264**		***n*=569**	
**Supraclavicular lymph nodes**	**Celiac lymph nodes**	**Liver**	**Lung**	**Sensitivity (%)**	**Specificity (%)**	**Sensitivity (%)**	**Specificity (%)**
CT	CT	CT	CT	66 (33/50)	95 (204/214)	68 (65/96)	96 (454/473)^a^
+USn: 1 pos	CT	CT	CT	86 (43/50)	95 (203/214)	84 (81/96)	96 (453/473)^b^
+USn: 1 pos	CT	+USa: 2 pos	CT	82 (41/50)	97 (208/214)	81 (78/96)	98 (463/473)^c^
+USn: 1 pos	CT	+USa: 2 pos	+CXR: 2 pos	78 (39/50)	99 (211/214)	74 (71/96)	99 (469/473)^d^

CT=computed tomography; CXR=chest X-ray; USa=ultrasound abdomen; USn=ultrasound neck.

a 569/569 values (100%) were present before imputation of missing values.

b 1115/1138 values (98%) were present before imputation of missing values.

c 1450/1707 values (85%) were present before imputation of missing values.

d 1874/2276 values (82%) were present before imputation of missing values.

**Table 5:** Costs, life expectancies, and quality adjusted life years (QALYs) for patients with oesophageal or gastric cardia cancer who had undergone all staging investigations (*n*=264)

**Supraclavicular lymph nodes**	**Celiac lymph nodes**	**Liver**	**Lung**	**Number of patients with operation (%)**	**Costs per patient (k$)**	**Life expectancy per patient (year)**	**QALY per patient**	**Δ Costs/Δ QALY (k$ per QALY)**
CT	CT	CT	CT	221/264 (84)	41.9	1.973	1.178	Dominated
+USn: 1 pos	CT	CT	CT	210/264 (80)	39.8	1.966	1.176	Reference
+USn: 1 pos	CT	+USa: 2 pos	CT	217/264 (82)	41.1	1.993	1.190	94,7
+USn: 1 pos	CT	+USa: 2 pos	+CXR:	222/264 (84)	42.0	2.010	1.198	113,3
	Surgery in all patients		2 pos	264/264 (100)	50.0	2.033	1.204	365,3

CT=computed tomography; CXR=chest X-ray; pos=positive scenario; USa=ultrasound abdomen; Usn=ultrasound neck.
